# Supplementary material for: Analysis of PPARγ Signaling Activity in Psoriasis
Source: Int J Mol Sci. 2021 Aug 10;22(16):8603. doi: 10.3390/ijms22168603 (PMC8395241; doi:10.3390/ijms22168603)
Supplement: Supplementary file 1 [file ijms-22-08603-s001.zip › Supplemental materials_Analysis of PPARg signaling activity in psoriasis/Pathway models/Models images and html files/PPARG inhibits phenotypes assotiated with psoriasis/210837.html]

endoplasmic reticulum stress


# Cell Process endoplasmic reticulum stress

|  |  |
| --- | --- |
| URN | urn:agi-cellproc:endoplasmic%20reticulum%20stress |
| Total Entities | 0 |
| Connectivity | 4579 |
| Name | endoplasmic reticulum stress |

---

|  |  |
| --- | --- |
| Pathway | New Pathway (2) |
|  | PPARG inhibits phenotypes assotiated with psoriasis |
|  | efects selected |
|  | 2\_Cigarette smoke and inflammation cause alveolar epithelial cell death in COPD |
|  | 3\_Autophagy dysfunction in Crohn's disease |
|  | 2\_Hyperglycemia and hyperlipidemia trigger beta-cell apoptosis |

---

|  |  |
| --- | --- |
| MedScan ID | 4206876 |

---

|  |  |
| --- | --- |
| Alias | endoplasmic reticulum stresses |
|  | endoplasmic reticulum stress |
|  | ER stress |
|  | ER stresses |

---
